# The Evaluation, Review, and Recommendations, Regarding Referrals to a West Yorkshire Specialist Forensic Community Team

**DOI:** 10.1192/bjo.2024.473

**Published:** 2024-08-01

**Authors:** Laura Gow

**Affiliations:** Cardiff University, Cardiff, United Kingdom. South West Yorkshire Foundation Trust, Wakefield, United Kingdom

## Abstract

**Aims:**

In the United Kingdom Specialist Community Forensic Teams (SCFT) are a new national development, aiming to enable and support earlier discharge from secure hospitals, and provide treatment to patients on a forensic pathway, in a community setting.

This project's ambition was to yield data to support future development of the service. The following research question was developed, as a focus for the project:

*‘In order for patients to be cared for as close to home as possible and for forensic services to reduce the length of inpatient stay, when should a patient be referred to a Specialist Forensic Community Team?’*

**Methods:**

The project was accepted by Cardiff University and South West Yorkshire Foundation Trust as a service evaluation.

The project methodology considered the impact of trauma throughout, given the forensic setting and high prevalence of trauma in individuals accessing forensic services.

A questionnaire was developed, which covered several relevant themes regarding the service, including the research question, and was distributed to patients, professionals, and referrers, either using, or associated with the team.

The total number of participants recruited was n = 45. These were made up of service users (n = 17), referrers to the service (n = 10), and other professionals (n = 18).

**Results:**

Participants felt they understood the purpose of the SCFT, placed importance on being involved in service evaluation, and were confident their responses would influence service development.

Reasons to refer to the SCFT were the perceived helpfulness of the team, supporting transitions, risk management, teamwork, therapeutic alliance, quality, and clinical knowledge.

Results favoured multi-disciplinary team agreement as being an important factor in the timing of SCFT referral. Upon admission, or granting of unescorted leave, were also cited as appropriate times to refer to the service. Clinically appropriate timing, individual needs, and service user motivation were additional indicators for SCFT referral.

**Conclusion:**

The West Yorkshire SCFT offers previously unavailable pathways from secure services into the community. The clinical model uses a trauma-informed, formulation-driven, collaborative approach to care, treatment, and risk management, which participants found favourable. Improved community pathways and connections offer a sense of improved hope, and a feeling of being helped, which is supportive of personal recovery.

There are recommendations which suggest that a community pathway agenda, embedded into services from admission, will support clinically appropriate timing of SCFT referral, and should be a decision which is made collaboratively, with patients, carers, and the multidisciplinary services around them.

